# Modern Sociology

**Published:** 1899-07-01

**Authors:** 


### Modern Sociology.

### THE STATE CHILDREN'S ASSOCIATION.

The State Children's Association has just issued its annual report. The work of this association has many sides, but one object, to secure the treatment of pauper children on individual rather than on institutional lines. To herd these children together in large "barrack" schools and block homes is the easiest way of dealing with them, but this is its only merit. It is not cheap. Witness the fact that it cost 10s. 7¾d. per week to maintain each child at the large schools at Banstead, and at Hornchurch £38 6s. 7d. per annum. One does not need to be a very expert statistician to see that such sums would cover the cost of maintaining a child in very different style from what it is desirable that pauper children should enjoy, or indeed than it is likely they do enjoy. It is not strange that the State Children's Association protest against the proposed expenditure of £72,000 for the formation of a pauper colony for 370 Mile End children. The wonder is that the ratepayers do not protest also. But the average ratepayer, though he must have his grumble, is, on the whole, a long-suffering animal. But the expense is the least evil. It has been demonstrated over and over again that children trained in institutions of any kind are not fit to cope with the world when they go out into it. This does not apply to pauper schools alone, but to all institutions where children are dealt with *en masse*. Even where some industrial training is given, it has been so thoroughly organised, so supervised, so drilled and controlled in every way, that the pupils have never had any training in the very thing that is most needful, the application of their own intelligence to unforeseen problems. Everything has been arranged for them; they have had to work, but not to think. But a training in observation, in estimating and dealing with the facts of everyday life, is worth more than a great deal of education in special handicrafts. This is what the boarded-out child gets; and this is why we have always recommended boarding-out for pauper children, even when it involves the child's lines being laid in places which are not pleasant. Of course the homes to which children are sent should be selected with care, and should be subject to frequent surprise visits; but the public conscience is now so much alive to the responsibility of dealing with these children of the State that there is no difficulty in finding guardians who will voluntarily undertake such work. Failing boarding-out in the homes of working people, it may be advisable to adopt a system of small special homes; but, in any case, the association advise that the children should go to the schools in the neighbourhood, should wear no distinctive dress, and should in no way be marked out from their fellows. In short, do not let the children feel that they are paupers, and they are not so likely to drift back to the workhouse in after life. The association also aims at bringing about a closer investigation of the circumstances and relations of poor-law children. If this were carefully done it is quite possible that the number to be maintained by the parish might be lessened, and in many cases it might be possible to find homes for the children elsewhere than under parish care. On the other hand, it is desirable to have further powers in order that the guardians may be able to deal with the children of tramps, and those wretched "ins and outs" who, in the midst of our civilisation, manage to be brought up as savages. Some children might be emigrated, for, under the new laws of Ontario and Manitoba, it is possible for the children sent there to be as well looked after and inspected as in Britain. But in every case the aim is to bring the children up, not as paupers, but as independent citizens, in whose memory there shall remain no idea of being maintained by charity, and who will therefore not be willing to revert to such maintenance, either for themselves or their children. The State Children's Association has suffered a great loss in the death of its President, Lord Herschell; but its work is so useful that it should be able to obtain a constantly-increasing measure of support from the public at large. Its offices are at 58, Old Bond Street. The hon. secretary is Mrs. Francis Rye.

### AN ILL-OMENED "MENAGE."

The word *menage* as used in Scotland has nothing to do with housekeeping; is, indeed, rather subversive to it, for it is the name given to a kind of lottery which is indulged in as an excitement by women of the working class. "It is resorted to," says a lady rent collector who has met with it in the course of her work, "to procure a pound of ready money, or, it may be, an article of dress or household furniture," more frequently, we think, the latter. A number of contributors subscribe a shilling or sixpence each, a number is drawn, and the one who draws the lucky number at once gets her pound. She is, of course, expected to go on paying up her subscription to the *menage* till all the other contributors have drawn their pounds. Weekly subscriptions are taken and weekly drawings made. The person who gets up the lottery charges a fee of threepence in the pound for her trouble—for every pound that is drawn, of course, so she does not fare badly. This woman is known as the manager—the *ménagère* from whom the lottery takes its name. Even when the lottery is conducted honestly it is far from being a good thing for those who participate in it. These weekly meetings to receive subscriptions and draw lots are usually inaugurated and finished with drinking, and are therefore demoralising, apart altogether from the gambling instinct to which they cater. But they are seldom honestly conducted. Before the twenty weekly meetings and drawings are past some of the contributors have left the neighbourhood. It is a peculiar fact that these are often those who have drawn the money in the earlier deals. Then there is much grumbling and recrimination, but no redress. It may be suspected that in some cases the *ménagère* has, for a consideration, connived at what is practically a robbery of her remaining clients. But even without dishonesty it may happen that one of the contributors cannot pay up her weekly instalment. In that case she loses all that she has put into the gamble. This kind of lottery is in vogue among working women in all parts of the kingdom, and is a thing which should be discouraged by all who have the interests of the poor at heart. At the best it is a dangerous method of raising money, and the surroundings in which it is conducted are for the most part more demoralising still.

